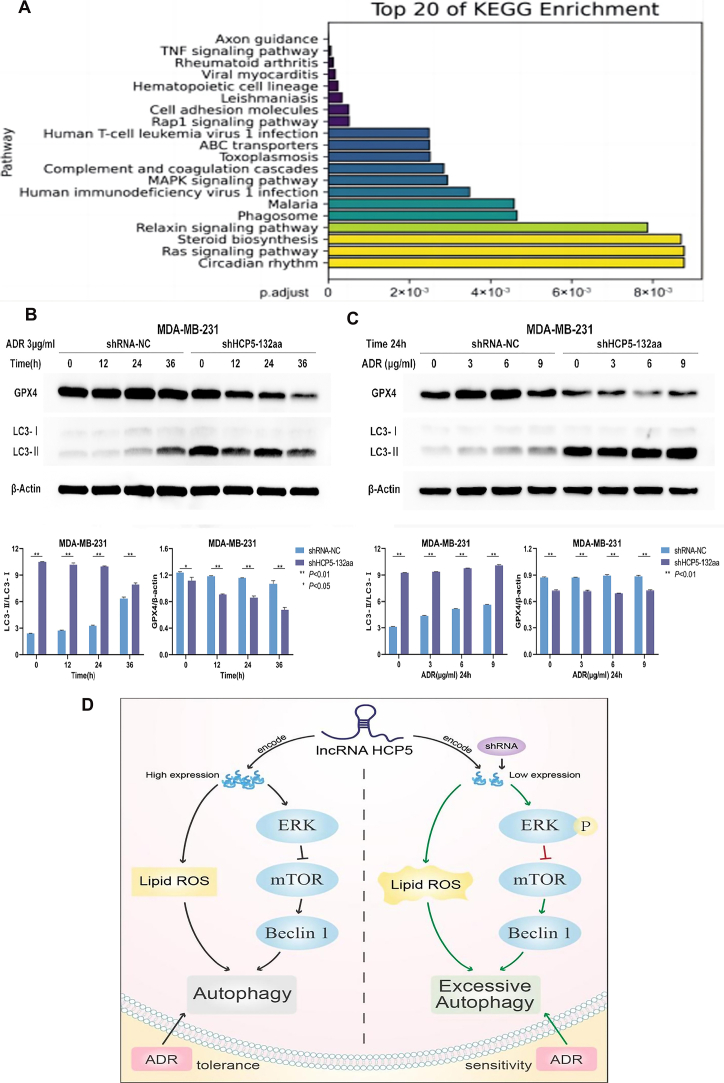# LncRNA HCP5-encoded protein contributes to adriamycin resistance through ERK/mTOR pathway-mediated autophagy in breast cancer cells

**DOI:** 10.1016/j.gendis.2023.06.002

**Published:** 2023-07-10

**Authors:** Jia-Ni Xing, Yi-Ni Shang, Zheng-Ling Yu, Shun-Heng Zhou, Wang-Yang Chen, Li-Hong Wang

**Affiliations:** a Department of Pathophysiology, Medical College of Southeast University, Nanjing, Jiangsu 210009, China; b Department of Biomedical Engineering, Nanjing University of Aeronautics and Astronautics, Nanjing, Jiangsu 211106, China; c School of Medicine, Taizhou University, Taizhou, Zhejiang 318000, China; d Jiangsu Provincial Key Laboratory of Critical Care Medicine, Nanjing, Jiangsu 210009, China

Adriamycin (ADR), also known as doxorubicin, is an anthracycline anticancer drug and a chemotherapeutic drug commonly used in breast cancer treatments.^1^ However, breast cancer patients can gradually become tolerant to chemotherapy. Therefore, improving the curative effect of ADR remains an urgent problem to be solved. Autophagy is a complex catabolic process; normal living cells break down damaged organelles or aggregated molecules and absorb energy to maintain homeostasis through autophagy. When autophagy dysfunction occurs, it will inevitably cause cell death. Long noncoding RNA (lncRNA) is a transcript longer than 200 nucleotides in length.^2^ As a human-specific gene, lncRNA human histocompatibility leukocyte antigen complex P5 (HCP5) is located in the major histocompatibility complex class Ⅰ region and can generate a transcript of approximately 2.5 kb. There is also a relationship between HCP5 and drug resistance.^3^

We have previously shown that the lncRNA HCP5 has the potential to encode a 132-amino acid protein which has been named HCP5-132aa by us.^4^ In this study, we aimed to investigate the possible involvement of HCP5-132aa in ADR resistance.

Our RT‒qPCR and Western blot analyses revealed that HCP5-132aa expression is significantly elevated in triple-negative breast cancer (TNBC) cells compared to other breast cancer subtypes. Moreover, we observed that the expression level of HCP5-132aa was markedly higher in the ADR-resistant MCF-7/ADR cell line than in its parental MCF-7 cells, as shown in Figure S1A and B. These findings suggest that HCP5-132aa expression is increased in both ADR-resistant breast cancer cells and TNBC cell lines.

To further investigate the impact of HCP5-132aa on breast cancer cells, we utilized lentivirus and plasmid transfection to generate cell lines with either knockdown or overexpression of HCP5-132aa. Among the four lentiviruses, we selected the one with the most effective knockdown for further experiments. We confirmed the efficiency of the transfection as seen in Figure S1C–F. HCP5-132aa overexpression was found to increase the rate of cell growth, as shown in Figure S1K.

Knockdown of HCP5-132aa using shRNA-1 and shRNA-2 inhibited cell proliferation and growth in both TNBC and MCF-7/ADR cells. Re-expression of HCP5-132aa using a transfecting plasmid reversed the growth rate, as shown in Figure S1G–I. In colony formation assays conducted with MCF-7/ADR and MDA-MB-231 cells, the number of colonies formed in the shRNA-1 and shRNA-2 groups was partially reduced compared with that of the control group, which was also recovered due to HCP5-132aa overexpression (Fig. 1A). As shown in Figure S1J, L, ADR exhibited more obvious inhibitory ability in the cells in the shHCP5-132aa group, which could be reversed by the autophagy inhibitor 3-methyladenine (3-MA). These findings confirmed the link between HCP5-132aa, cell sensitivity to ADR, and autophagy.

**Figure 1:**
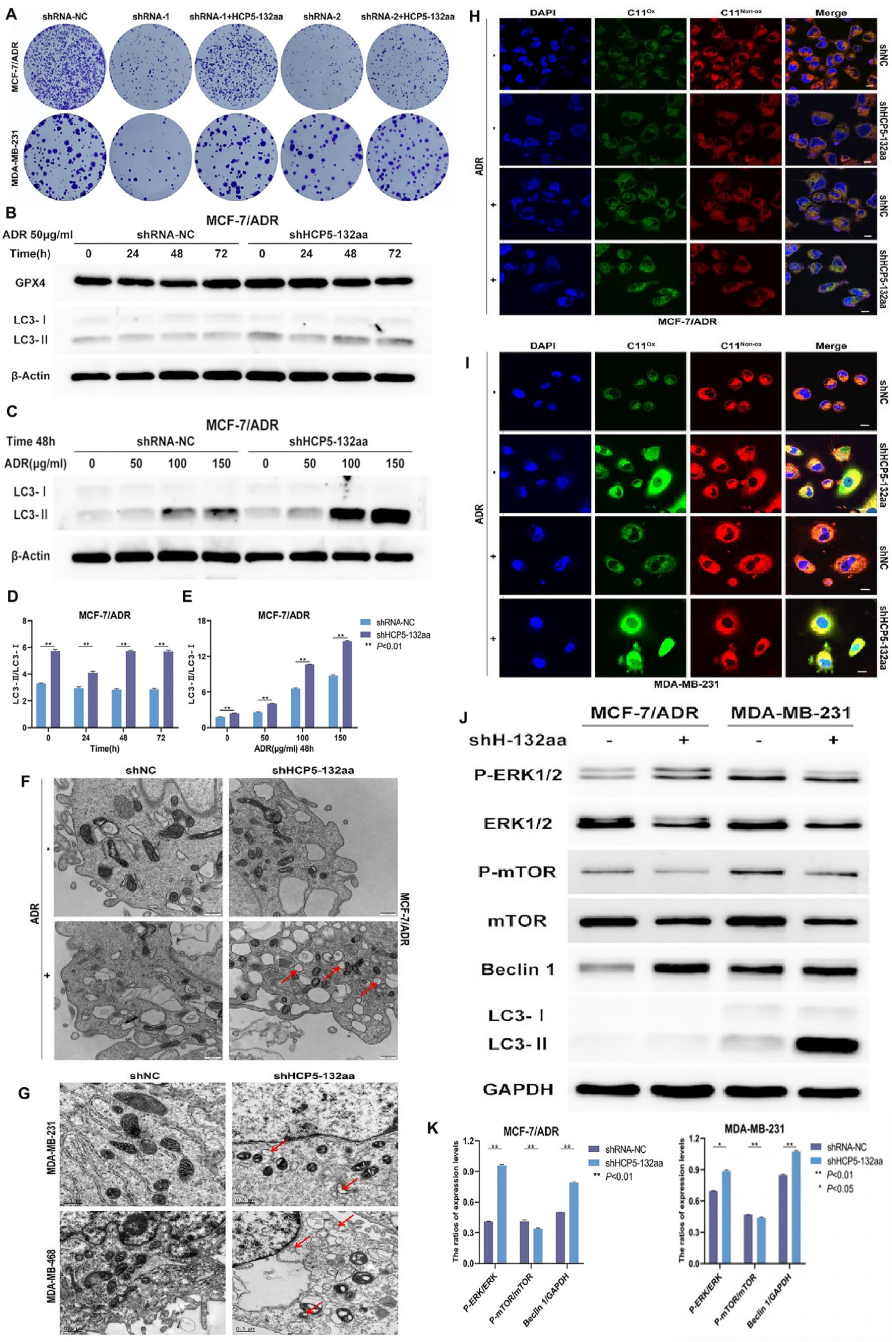
HCP5-132aa promotes resistance of breast cancer cells to ADR through ERK/mTOR pathway-mediated autophagy. **(A)** The colony formation results in MCF-7/ADR and MDA-MB-231 cells with or without HCP5-132aa. **(B)** The expression levels of LC3 and GPX4 in MCF-7/ADR cells treated with ADR for 0, 24, 48, and 72 h. **(C)** The expression level of LC3 in MCF-7/ADR cells treated with ADR at the concentration of 0, 50, 100, and 150 μg/mL. **(D**, **E)** The quantification results of LC3 expression level in (A, B). **(F)** The accumulation of vacuoles was observed after ADR stimulation in MCF-7/ADR cells with or without HCP5-132aa. Scale bar: 0.5 μm. **(G)** The accumulation of vacuoles was observed after knocking down HCP5-132aa in MDA-MB-231 and MDA-MB-468 cells. Scale bar: 0.5 μm. **(H**, **I)** The content of lipid ROS in the shHCP5-132aa and control cells treated with or without ADR was detected using laser confocal microscopy in MDA-MB-231 and MCF-7/ADR cells (1000×). Blue: nucleus; green: oxidation state; red: non-oxidation state. Scale bar: 10 μm. **(J**, **K)** The expression levels of ERK1/2, mTOR, Beclin 1, and LC3 in MCF-7/ADR and MDA-MB-231 cells. The data were presented as mean ± standard deviation (*n* ≥ 3). Significance in (D, E, K) was tested by Unpaired student's *t*-test.

We then investigated the impact of HCP5-132aa knockdown on the sensitivity of breast cancer cells to ADR treatment. To test this hypothesis, we treated MCF-7/ADR and MCF-7 cells with increasing doses of ADR while assessing the influence of HCP5-132aa. Our CCK-8 assay results indicated that the inhibition rate of the HCP5-132aa knockdown group was higher in drug-resistant MCF-7/ADR cells. Notably, the IC_50_ of ADR in MCF-7/ADR cells cultured for 48 h was 306.4 μg/mL, whereas the IC_50_ in MCF-7 parental cells was only 15.74 μg/mL, as shown in Figure S1M–T. These results further demonstrate the resistance of MCF-7/ADR cells to ADR and suggest that HCP5-132aa knockdown can increase the sensitivity of these cells to ADR.

During autophagy, cytoplasmic LC3-Ⅰ undergoes a small segment degradation process to form enveloped LC3-Ⅱ. The ratio of LC3-Ⅱ to LC3-Ⅰ is often used as an indicator of autophagy. In MCF-7/ADR cells cultured with ADR, the HCP5-132aa knockdown group exhibited higher levels of dose- and time-dependent autophagy (Fig. 1B–E).

Autophagy is a crucial factor that affects chemoresistance in breast cancer, and the gold standard for its detection is the observation of cell ultrastructure under a transmission electron microscope (TEM). We observed distinct vacuole accumulation in HCP5-132aa-knockdown MCF-7/ADR cells treated with ADR, indicating increased autophagy (Fig. 1F, G). The TEM results confirmed that HCP5-132aa knockdown promoted autophagy. In addition, the merged confocal images showed the fluorescence intensity of MDA-MB-231 and MCF-7/ADR cells in the shHCP5-132aa group, indicating greater production of lipid reactive oxygen species (ROS) (Fig. 1H, I).

Furthermore, we conducted RNA sequencing on both control and HCP5-132aa-knockdown MDA-MB-231 cells and identified 720 differentially expressed genes (DEGs). KEGG pathway enrichment analysis of the DEGs revealed the top 20 enriched pathways, including the Ras signaling pathway, the ATP-binding cassette (ABC) transporter pathway, and the mitogen-activated protein kinase (MAPK) signaling pathway, all of which are associated with drug resistance (Fig. S2A). Western blot analysis showed that HCP5-132aa knockdown in MCF-7/ADR and MDA-MB-231 cells not only increased the ratio of LC3-II to LC3-I but also induced ERK phosphorylation and inhibited mTOR activity, leading to increased expression of the downstream autophagy molecule Beclin 1 (Fig. 1J, K). These findings suggest that HCP5-132aa may regulate autophagy in breast cancer cells through the ERK/mTOR pathway, thereby affecting drug resistance.

Based on previous studies suggesting a relationship between autophagy and ferroptosis, we also measured the levels of glutathione peroxidase 4 (GPX4), a key regulator of ferroptosis. However, after HCP5-132aa knockdown, there was no significant difference in GPX4 levels, and the ratio of LC3-II to LC3-I remained constant over time in drug-resistant MCF-7/ADR cells (Fig. 1B). It seemed that HCP5-132aa did not affect MCF-7 cell resistance to ADR through ferroptosis. However, in MDA-MB-231 cells, the expression of GPX4 and the ratio of LC3-II to LC3-I decreased over time in the shHCP5-132aa group. However, these changes were not affected by alterations in the concentration of ADR (Fig. S2B, C). It seemed that more ferroptosis and less autophagy occurred over time in HCP5-132aa-knockdown TNBC cells treated with ADR.

In conclusion, we determined that the protein HCP5-132aa, encoded by lncRNA HCP5, plays a tumor-promoting role and is highly expressed in MCF-7/ADR and TNBC cell lines. Decreased expression of HCP5-132aa can elevate the accumulation of lipid ROS and enhance the level of the autophagy regulatory protein Beclin 1 in ADR-treated cells through the ERK/mTOR pathway, thus inducing excessive autophagy (Fig. S2D). The results suggest that the lncRNA-encoded products can be used in tumor chemotherapy for better effects. However, due to limited research on the function of lncRNA coding products, more studies need to be conducted to confirm these findings.

## Author contributions

Conceptualization, L.-H.W.; methodology, J.-N.X. and Y.-N.S.; validation, Z.-L.Y.; formal analysis, S.-H.Z.; investigation, J.-N.X.; resources, Y.-N.S.; data curation, J.-N.X.; writing-original draft preparation, J.-N.X.; writing-review and editing, L.-H.W.; visualization, J.-N.X.; supervision, W.-Y.C.; data analysis, L.-H.W.; project administration, L.-H.W.; funding acquisition, L.-H.W. All authors have read and agreed to the published version of the manuscript.

## Conﬂict of interests

The authors declare no conflict of interests.

## Funding

This research was funded by the 10.13039/501100001809National Natural Science Foundation of China (No. 81972478), the Fundamental Research Funds for the Central Universities (China) (No. 2242020K40131), and the Jiangsu Provincial Key Laboratory of Critical Care Medicine (China) (No. JSKLCCM-2022-02-013).

## Figures and Tables

**figs1:**
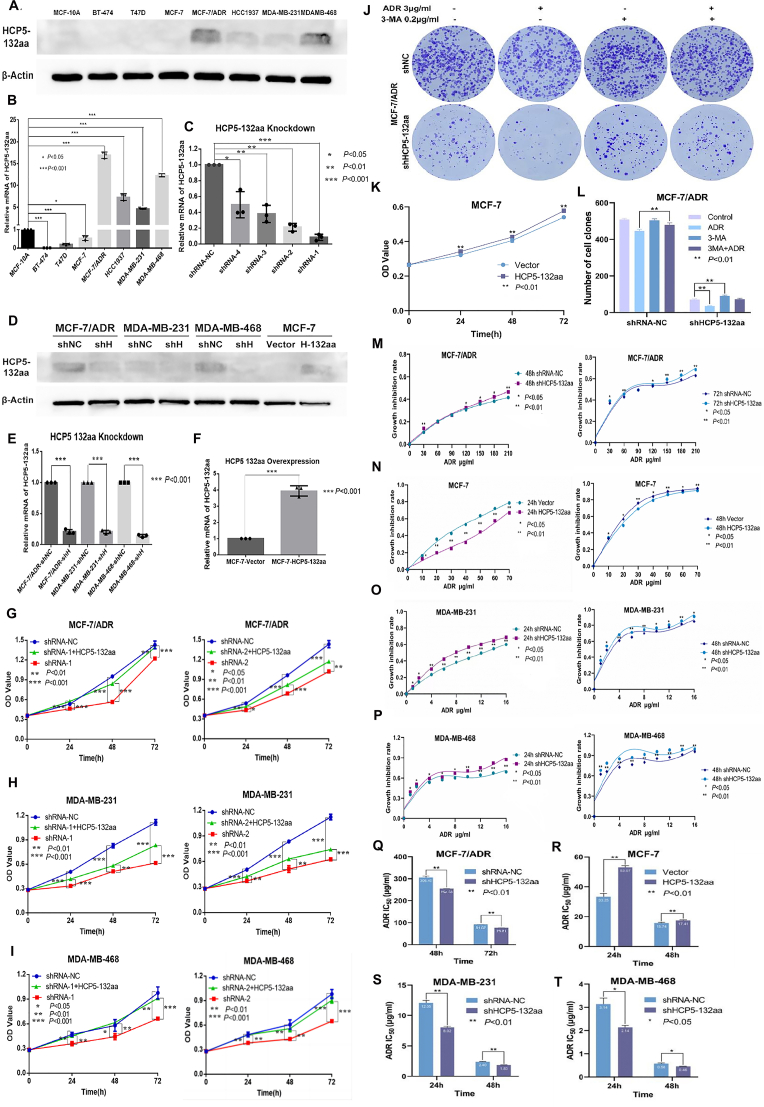


**figs2:**