# Supplementary material for: LncRNA HCP5-encoded protein contributes to adriamycin resistance through ERK/mTOR pathway-mediated autophagy in breast cancer cells
Source: Genes Dis. 2023 Jul 10;11(4):101024. doi: 10.1016/j.gendis.2023.06.002 (PMC10937560; doi:10.1016/j.gendis.2023.06.002)
Supplement: Multimedia component 1 [file mmc1.docx]

**Supplementary Data 1**

Materials and Methods

**Cell culture**

MDA-MB-231 and MDA-MB-468 cell lines (obtained from the Shanghai Institutes for Biological Sciences, Chinese Academy of Sciences Shanghai) were grown in Leibovitz’s L-15 medium (BasalMedia, China) containing 10% heat-inactivated fetal bovine serum (FBS) (Biological Industries, Israel), in a carbon dioxide-free atmosphere at 37℃. The MCF-7/ADR cell line (donated by Harbin Medical University Cancer Institute, China) was grown in 1640 medium (Gibco, USA) containing 10% FBS, in a 5% carbon dioxide atmosphere at 37℃. The MCF-7 cell line (obtained from the Shanghai Institutes for Biological Sciences, Chinese Academy of Sciences Shanghai) was grown in MEM medium (Gibco, USA) containing 10% FBS, sodium pyruvate, non-essential amino acids, glutamine and insulin, in a 5% carbon dioxide atmosphere at 37℃. In an ADR-related experiment, cells were cultured under original conditions after adding different concentrations of ADR (Selleck, China) to the medium.

**Transfection**

Breast cancer cells were seeded in 6-well plates and incubated overnight. After replacing with fresh medium, appropriate amounts of lentivirus were added to the plates containing the cells. Cells were cultured in a fresh renewal medium after 48 hours. Fluorescence microscopy and RT-qPCR were used to verify the transfection effect. The lentivirus (GenePharma, China) contained the shRNA-NC group (empty vector) and shHCP5-132aa group with the HCP5-ORF-shRNA sequence (shRNA-1 5'-GGACGATTCTCCTCACACTTA-3'; shRNA-2 5'-GGATCTATTACCTGTGCCTGG-3'; shRNA-3 5'-GAACTCCTCCTACCCTCATTG-3'; shRNA-4 5'-GGTCTGGGCTCTTGGAAATCA-3').

**RT-qPCR analysis**

The RNA was extracted from cells using the Trizol method and transcribed into complementary DNA (cDNA) by reverse transcriptase polymerase chain reaction. Then, the qPCR was carried out with cDNA, primers (Sangon Biotech, China) and ChamQ SYBR qPCR Master Mix (Vazyme, China). The following primers were used as follows: HCP5-132aa forward, 5'-TGGCTGGACGATTCTCCTCACAC-3' and reverse 5'-GAGGCATGGCTGCTGTCACAC-3'; GAPDH forward, 5'-CGGAGTCAACGGATTTGGTCG-3' and reverse 5'-TCTCGCTCCTGGAAGATGGTGAT-3' and the expression levels were normalized to the control.

**Colony formation assay**

The 6-well plates were spread with MCF-7/ADR or MDA-MB-231 cells (800/well). During the 3-week period of culture under normal conditions, the medium was changed periodically over time to ensure the normal growth of cells. When the clone was formed, the steps below were followed. The medium was discarded, and the plates were washed twice with PBS. A total of 1 ml/well of methanol were added to the plates for cleaning up. Then, to each well was added 1 ml of 0.1% crystal violet, which was used for dying for 1 hour. The plates were washed and photos were taken after air drying.

**Transmission electron microscopy**

After the centrifugation of the TNBC cell suspension, cell precipitates were collected and fixed in 2.5% glutaraldehyde at 4 ℃ overnight. The cell precipitates were rinsed with phosphate buffer and 1% osmic acid and dehydrated with different concentrations of acetone. The soaked sample was wrapped in the embedding agent and cut into ultra-thin pieces. The pieces were sent to be photographed under the transmission H7500 electron microscope (Hitachi, Japan).

**Lipid reactive oxygen species assay**

The TNBC cells were seeded in Φ20 mm glass-bottom dishes (Nest, China) overnight, and the medium was replaced with ADR diluent for 24 hours. The medium with ADR was discarded, and to each cell culture dish was added 1ml HBSS (Gibco, USA), 3ul 1mg/ml BODIPY 581/591 C11 (Invitrogen, USA) and 0.5 ul 10 mg/ml Hoechst (Invitrogen, USA) mixed dye, and then incubated at 37 ℃ without CO2 for 10 minutes. The dye was removed, and 2 ml of HBSS was added to each dish. The dishes were observed using an Olympus Fluoview 1000 confocal microscope and photographed with fluorescence.

**CCK-8 assay**

MDA-MB-231 cells were added to 96-well plates and attached for 24 hours. The original medium was replaced with the drug mixture medium. The 96-well plates were added with CCK-8 solution and cultured at 37 ℃ for 3 hours. The enzyme labeling instrument was then used to detect the OD value. The data were organized to calculate the cell inhibition rate.

**Western blot**

The cells with ADR application were lysed and collected to centrifuge at 10000 rpm for 10 minutes at 4 ℃. The supernatant served as the protein lysate and was stored at 80 ℃. The total protein concentrations were determined by using the BCA protein assay kit (Beyotime, China). Then, the same amounts of protein were subjected to sodium dodecyl sulfate polyacrylamide gel electrophoresis (Beyotime, China) and transferred to polyvinylidene fluoride membranes (Millipore, USA). After blocking with 5% skimmed milk, the membrane was put into the diluent which contained the primary antibodies, including HCP5 (1:2000, ImmunoWay, #YN4243, USA), LC3 (1:2000, Proteintech, USA), extracellular signal-regulated kinase 1/2 (ERK1/2), Phospho-ERK1/2 (P-ERK1/2), mammalian target of rapamycin (mTOR), Phospho-mTOR (P-mTOR) (1:1000, Cell Signaling Technology, USA), Beclin 1 (1:4000, Proteintech, USA), GAPDH (1:20000, Proteintech, USA) and β-actin (1:10000, Proteintech, USA), at 4 ℃ overnight. The membranes were washed three times with TBST and treated with secondary antibody (1:10000, Biosharp, China) in 5% skimmed milk for 1 hour at room temperature. Targeted protein bands on membranes were photographed with the luminescent image analyzer after using chemiluminescent substrate. The photograph was analyzed using the Image J software.

**Coding prediction tools**

The full-length sequence FASTA format of lncRNA HCP5 was obtained from the National Center for Biotechnology Information (https://www.ncbi.nlm.nih.gov/nuccore/ NC_000006.12?report=fasta&from=31463180&to=31465809). The HCP5 sequence was searched using the online tools ORFfinder (https://www.ncbi.nlm.nih.gov/orffinder/) , CPAT (http://lilab.research.bcm.edu) and CPC2 (http://cpc2.gao-lab.org/index.php). High-throughput sequencing technologies, including ribosome profiling (Ribo-seq) and tandem mass spectrometry (MS/MS), were used to verify the lncRNA HCP5- encoded products in the MDA-MB-231 cell line. To screen candidate ORFs, the Ribo-seq data (GSE69923) were downloaded from the Gene Expression Omnibus database. Then, the Ribo-seq files in SRA format were converted to FASTQ format, using the fastq-dump tool (https://ncbi.github.io/sra-tools/fastq-dump.html). Next, the Trim Golare (https:// www.bioinformatics.babraham.ac.uk/ projects/ trim_ galore/) and RNAcentral database were separately used to trim adaptor sequences and filter ribosomal RNA (rRNA) sequences. Afterwards, BAM format files was obtained by using TopHat2, which aligned the remaining reads to the reference genome GRCh38. Finally, the MS/MS data (PXD008222) were downloaded from the EMBI-EBI-PRIDE database and converted to MGF format using MSConvert. The sequences of peptides or proteins were aligned to GRCh38 using Peppy, which confirmed the HCP5 ORF with MS/MS peptides mapped. The Deseq2 calculated differentially expressed genes, and the clusterProfiler performed pathway enrichment analysis.

**Statistical analysis**

All the statistical analysis was performed by the IBM SPSS Statistics 23.0 software and GraphPad Prism 8. Kruskal-Wallis test and One-way ANOVA analysis with Dunnet’s post hoc and Kukey’s multiple comparisons tests were used to compare the differences of multiple groups. Two mean values comparison were performed by Unpaired Student's *t*-test. **P* < 0.05 indicated statistical significance. The results were then processed with GraphPad Prism 8 to be formatted as visual figures.

**Supplementary Data 2**

**Fig S1. HCP5 regulates autophagy to promote proliferation and resistance to ADR in breast cancer cells. (A)** The results of expression level of HCP5-132aa from the Western blot. **(B)** The results of expression level of HCP5-132aa from the RT-qPCR. **(C)** RT-qPCR results of HCP5-132aa knockdown. **(D)** Western blot results of HCP5-132aa knockdown or overexpression. **(E-F)** RT-qPCR results of HCP5-132aa knockdown or overexpression. **(G)** The OD values of MCF-7/ADR cell proliferation with or without HCP5-132aa. **(H)** The OD values of MDA-MB-231 cell proliferation with or without HCP5-132aa. **(I)** The OD values of MDA-MB-468 cell proliferation with or without HCP5-132aa. **(J)** The colony formation results in MCF-7/ADR cells treated with ADR or 3-MA. **(K)** The OD values of MCF-7 cell proliferation with HCP5-132aa. **(L)** The quantification results of Figure S1J. **(M-P)** The inhibitory rate of ADR in MCF-7/ADR, MCF-7, MDA-MB-231 and MDA-MB-468 cells without HCP5-132aa treated for 24 or 48 h. **(Q-T)** The IC50 of ADR in MCF-7/ADR, MCF-7, MDA-MB-231 and MDA-MB-468 cells without HCP5-132aa treated for 24 or 48 h. Data was presented as mean ± standard deviation, n ≥ 3, Significance in **(B)** was calculated using the Kruskal-Wallis test with Dunn’s multiple comparisons test. Significance in **(C)** was calculated using the one-way ANOVA with Dunnett’s multiple conparisons test. Significance in **(E), (F), (K)** and **(M-T)** was calculated using the Unpaired student’s *t*-test. Significance in **(G-I)** and **(L)** was calculated using the one-way ANOVA with Kukey’s multiple comparisons test.

**Supplementary Data 3**

**Fig S2**. **The expressive levels of LC3 and GPX4 in breast cancer cells treated with ADR.** **(A)** The summary of the top 20 enriched pathways from 720 differentially expressed genes, which were obtained from HCP5-132aa knockdown MDA-MB-231 cells. **(B-C)** The expression levels of LC3 and GPX4 in MDA-MB-231 cells treated with ADR for 0, 12, 24 and 36 h or at the concentration of 0, 3, 6 and 9 μg/ml. **(D)** Autophagy mechanism affected by knocking down the expression of lncRNA HCP5-encoded protein. Data was presented as mean ± standard deviation, n ≥ 3, Significance in **(B)** and **(C)** was calculated using the Unpaired student’s *t*-test.
